# Palaeoecological deductions from osteohistology

**DOI:** 10.1098/rsbl.2023.0245

**Published:** 2023-08-23

**Authors:** Anusuya Chinsamy

**Affiliations:** Department of Biological Sciences, University of Cape Town, John Day Building, University Avenue, Rondebosch 7700, South Africa

**Keywords:** palaeohistology, bone histology, microanatomy, palaeoecology

## Abstract

Palaeoecological deductions are vital for understanding the evolution and diversification of species within prehistoric environments. This review highlights the multitude of ways in which the microanatomy and microscopic structure of bones enables palaeoecological deductions. The occurrence of growth marks in bones is discussed, and their usefulness in deducing the ontogenetic status and age of individuals is considered, as well as how such marks in bones permit the assessment of the growth dynamics of individuals and species. Here osteohistology is shown to provide insight into the structure of past populations, as well as ecological relationships between individuals. In addition, the response of bones to trauma, disease and moulting is considered. Finally, I explore how osteohistology can give insight into ecomorphological adaptations, such as filter feeding, probe feeding and saltatorial locomotion. Methodological advances in three-dimensional microtomography and synchrotron scanning bodes well for future studies in osteohistology and despite some compromises in terms of tissue identity, circumvents the crucial issue of destructive analyses.

## 1. Introduction

Palaeoecology uses fossils and other proxies to reconstruct prehistoric species and communities within past ecosystems [1]. It is firmly integrated within the field of ecology and enables answers to important questions such as the origin of current biodiversity, communities within ecosystems, adaptation to prevailing environmental conditions, as well as helps to provide explanations regarding the biogeographic distribution of extant species. A cornerstone of palaeoecological studies is the idea of ecological uniformitarianism which suggests that natural processes operating in the past are basically the same as ones operating in the present [1]. Ecological uniformitarianism stems from James Hutton's original idea of uniformitarianism which is summarized in the statement that ‘the present is the key to the past’.

Palaeoecological studies permit a deep time perspective that allows an assessment of how ecological communities respond to change in the distant past, and often permits rigorous predictions regarding their responses in future especially under the threat of global environmental changes. Thus, data gathered from past ecosystems enable better estimations of predictions for future ecosystems.

Our knowledge of past communities of organisms derives from their preserved remains, i.e. fossils. There are a wide range of different types of fossils that have been recovered from the palaeontological record. For example, the Precambrian ‘Ediacaran’ biota preserves impressions of multicellular soft-bodied organisms that have allowed the reconstruction of some of the earliest ecosystems and show us the rich biodiversity that existed at that time some 635–538 Ma [2]. The slightly younger Burgess shale fossils are mainly preserved as impressions, but some with hard tissue preservation have given us a glimpse into the rich benthic ecosystems that existed on our planet about 508 Ma [3]. Younger fossil deposits show exquisite plant and insect fossils that give us an exceptional account of the floral diversity and insect radiation that occurred. There are many examples of insect damage on the fossil leaves that gives clues as to their biotic interactions [4], and in some instances unique insight into plant behaviour, such as nyctinasty [5]. Using the fossilized pollen and trackways that were preserved in a locality in Yorkshire (UK) Slater *et al.* [6], reconstructed the vegetation that existed in the area, and showed that this Middle Jurassic ecosystem consisted of bryophytes, lycophytes, ferns and a number of gymnosperms such as Araucariaceae and other giant conifers. The preserved dinosaur footprints suggest that the environment provided a rich food source for both large sauropod-size dinosaurs, as well as small, bipedal herbivorous dinosaurs. Using the fossil pollen recovered over a stratigraphic sequence these researchers were also able to show how the plant groups changed over time. Even trace fossils, such as fossilized tracks and trackways have been an incredibly rich source enabling the reconstruction of past communities, as well as glimpses into their behaviour (e.g. running or swimming dinosaurs), e.g. [7], and in some cases even stampedes and predation (e.g. [8]).

Many hard parts of vertebrates i.e. their bones and teeth, are preserved in the fossil record. The resilience of vertebrate skeletons in the fossil record is because bones and teeth are composite tissues that are made up of both organic and inorganic (mineral) components (e.g. [9]). Like other soft tissues, the organic parts of bones and teeth (like blood vessels, nerves, etc.) decompose soon after the animal dies, but the bones and teeth often survive in the fossil record because the minerals (the apatite or carbonated calcium phosphate) remain virtually intact by undergoing only minor ångström level changes. Since the organic and inorganic parts of bones and teeth are so intimately associated during life, even though the organic tissue decomposes, the inorganic tissue preserves their structural organization (e.g. [9]). However, not all bones that are buried survive into the fossil record. There are various taphonomic processes that affect an animal from the time it dies to burial, and even the geochemistry of the depositional environment affects the preservation of bones. Often, during fossilization, even the hard parts of organisms are completely obliterated (which explains the many gaps in our understanding of biotic change over time). There are also instances where although the morphology of the fossil bone is preserved, the microscopic structure is diagenetically altered by the infiltration of minerals (e.g. [9]), or by invading microbes (e.g. [10–13]).

When the mineralized hard parts of an animal survive into the fossil record, they provide a huge amount of information about the extinct animal; depending on what is preserved, the entire skeleton can be reconstructed, and if the fossil assemblage preserves the remains of other animals, a host of deductions can be made regarding the ecological community, as well as possible interactions between animals. Monospecific assemblages often indicate social groups, conspecifics or broods, whereas multiple species in the deposit could indicate predators and prey, etc. Besides providing much information regarding the species community and overall ecology of a locality, fossilized bones are also enormously useful in helping to reconstruct what the animal looked like, as well as enabling various functional attributes such as how it moved, stood or what it ate. If bones of different-sized animals are preserved in the assemblage, deductions about morphometric changes through ontogeny can be deduced. But this is not all. The microanatomy and microstructure of fossil bones and teeth give us much more information about the biology and life history of extinct animals. The focus of this review is on bone histology but note that teeth histology is also an exceptional resource to assess life-history traits, such as the age of weaning (in the case of mammals) and attainment of sexual maturity. Indeed, used in combination, bone and teeth histology offers unparalleled insights into the palaeobiology of extinct animals (e.g. [14,15]). Note that although valuable contributions have been made on the mineralized tissues of extant primates which has involved a range of topics on the biology of bone (e.g. [16,17]), as well as on incremental markings in dental tissues (e.g. [14]), this review emphasizes non-primate osteohistology. The aim of this review is to show how the osteohistology of well-preserved, microbially unaltered fossil bone permits the reconstruction of various ecological/environmental parameters of extinct animals. A selection of histological variables such as bone microanatomy, growth marks in bone, sex-specific bone tissues, the response of bone to trauma, disease, and moulting, as well as examples of some ecomorphological osteological correlates are discussed to demonstrate their contributions to palaeoecology.

## 2. Bone microanatomy

The internal architecture and compactness of a bone, i.e. its microanatomy, provides a huge amount of information about the lifestyle adaptations of the animal. It is well recognized that terrestrial animals have relatively thick long bone walls, with a central medullary cavity that is free of any bone. Such tubular bone structures are thought to be an adaptation for the biomechanical stresses and strains caused by walking on land [18]. Curiously, even thicker cortical bone walls have been recognized in burrowing animals as an adaptation for their fossorial lifestyles (e.g. Chinsamy & Hurum [19], Montoya-Sanhueza & Chinsamy [20]). In a study that examined the microanatomy of the largest members of the iconic family of African mole-rats (Bathyergidae), the Cape dune mole rat, *Bathyergus suillus*, Montoya-Sanhueza & Chinsamy [20] showed how the bone thickened during ontogeny because of the deposition of endosteally and periosteal formed bone tissue and reduced levels of endosteal resorption and secondary bone remodelling. Similar endosteal thickening through ontogeny has been documented in other fossorial animals (e.g. naked mole-rats [21]; aardvarks, *Orycteropus afer* [22]; wombats [23], as well as for *Chersina angulate* (the angulate tortoise) [24]).

By contrast, flying tetrapods, such as birds, pterosaurs and bats have thin-walled tubular bones, e.g. [25]. Aquatic birds, such as penguins, as well as other diving birds, such as loons and grebes, have much thicker bone walls as compared to their volant relatives, to overcome buoyancy in water (e.g. [26]). The microanatomy of aquatic animals is strikingly different to that of terrestrial animals. A glance at their cross sections shows a bony tissue that extends deep into the medullary cavity; the bone wall is often dense, i.e. pachyosteosclerotic (with reduced medullary cavities) in shallow water divers/slow swimmers, whereas deep divers and fast swimmers, like cetaceans, have a highly spongy bone tissue termed osteosclerotic (e.g. [27]).

There have been several studies that have focused on the bone microanatomy of the bones of extinct animals to deduce their possible lifestyles, and swimming modes (e.g. see [28]). For example, adaptations for a burrowing lifestyle have been deduced for *Cistecephalus* [29], while placodonts (e.g. [30]) and ichthyosaurs (e.g. [31]) show adaptations for aquatic lifestyles, with placodonts possibly adapted to shallow marine habitats, and ichthyosaurs, like dolphins for fast swimming and deep diving lifestyles [32].

A study of the tarsometatarsi of eight Eocene penguin species from Antarctica showed significant variation in the microanatomy of their bones [33]. Some of them had distinctly osteosclerotic bones, whereas others had well-developed medullary cavities. Based on the microanatomical variations, Cerda *et al.* [33] proposed that the penguins were adapted to different lifestyles i.e. the ones with dense osteosclerotic bone with reduced or absent medullary cavities were likely adapted for diving to deeper depths.

## 3. Bone microstructure

The microscopic structure of bones provides a huge amount of information regarding various aspects of the biology of the animal, particularly in terms of growth and life-history traits (e.g. [9,34,35]). For example, the organization of the collagen fibres in bone, and the type and extent of vascularization present is a direct reflection of the rate at which the bone was deposited, whereas the nature and texture of the bone provides information regarding various aspects of the life history of the animal. Depending on the type of bone tissue that is present, the growth dynamics of the animal can be deduced, as well as other information such as when hatching [36]) or birth [37] occurred, and the attainment of sexual and skeletal maturity (e.g. [38]). Areas in the bone subjected to repeated biomechanical stresses and strains are also indicated (e.g. [38]). Often deviant bone tissue types, i.e. abnormal bone tissue, are indicative of some kind of trauma or disease that afflicted the animal (e.g. [39]), and provide an unprecedented view of the life experiences of the animal. Histological studies of the bones of modern animals with known life-history data, particularly within the framework of the extant phylogenetic bracket are crucial to ensure that extrapolations to extinct animals are reliable and meaningful (e.g. [39,40]).

### (a) Growth marks in bone: age and growth curve analyses

**Figure 1.**
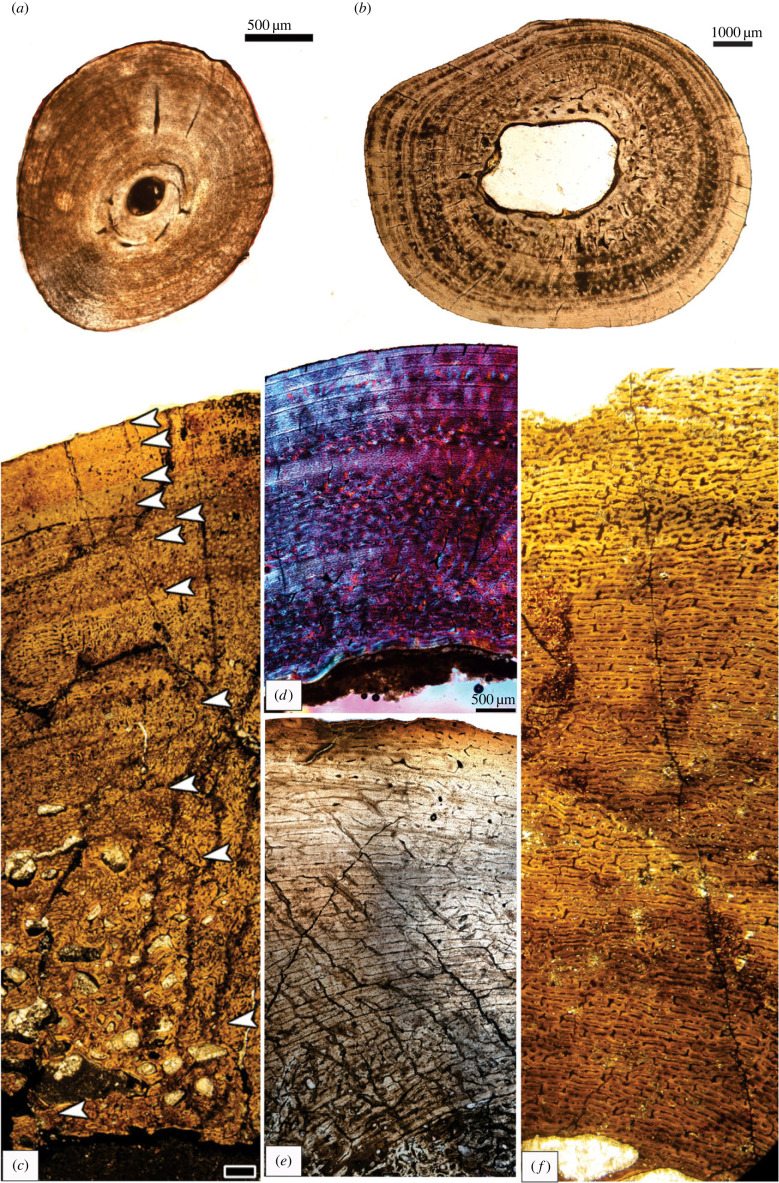
Growth marks evident in the compacta of (*a*) *Chersina angulata* femur (fm 20), (*b*) *Caiman latirostris* tibia, (*c*) *Mussaurus* femur (MLP68-11-27), (*d*) *Macropus fuliginosus* femur, KNW 6, (*e*) aepyornithid femur (046) and (*f*) absence of growth marks in *Mussaurus* femur MLP68-11-26-1.

Periodic growth spurts (zones) and periods of slowed growth (annuli), as well as stoppages in growth (lines of arrested growth) are often reflected in the bones of vertebrates as growth marks (figure 1). Like tree rings, the growth marks can be counted to obtain an estimate of the age of the animal, although one needs to be cognizant of bone remodelling and reconstruction that may obliterate earlier growth marks [9]. Cyclically formed bone tissues are ubiquitous among ectotherms whose growth is seasonally affected (figure 1*a*,*b*), but they are also increasingly recognized in the bones of endothermic mammals: for example, monotremes [19], marsupials (figure 1*d*) [19,38,41], eutherians such as polar bears [42], deer [43], equids [15], including rodents, which are the most speciose mammalian clade. Although modern birds grow up rapidly within a few weeks to months, the kiwi, (*Apteryx*) shows regular cyclical patterns of bone deposition throughout ontogeny [44]. Growth marks have been documented in several Mesozoic birds [7], as well as in several giant extinct birds (e.g. [45,46]), as well as in some large flightless ratites [47,48]. Although in the latter case, it must be noted that these samples were all from captive birds subjected to temperatures outside their adaptive range. It is, however, noteworthy that when confronted with unfavourable conditions these modern large captive birds were able to adopt a flexible developmental strategy, similar to their dinosaurian ancestors [47,49].

A count of the growth marks preserved in the bones of various extinct and extant vertebrates have been used to estimate the age of the animal, and have been used to reconstruct life-history traits such as ontogenetic age, age at maturity, growth rate, growth dynamics, etc. (e.g. [50–56]). Studies using fluorochrome staining have documented that an annulus and a zone are formed annually in crocodiles (see [9]) and age data through skeletochronology has been validated for several other taxa of known age (e.g. [57–59]). However, there are a few studies on known aged animals which suggest that the situation is much more complex than previously realized, and that a count of the growth marks does not accurately reflect the known ontogenetic age of some animals. For example, Schucht *et al.* [40] examined the growth marks in several known aged, captive, tetrapods using decalcified and undecalcified thin sections. They reported several ambiguities between the growth mark count in the same bones using different methodological approaches. In the study of multiple bones of the known aged, bearded dragon, Teixeira [59] found that only the tibia preserved growth marks that accurately reflected the real age of the specimen, whereas other bones of the skeleton underestimated the age of the individual. More recently, Janello & Chinsamy [60] reported the presence of multiple growth marks in juvenile giraffes which implied that they could not be annually formed, but rather likely reflected catastrophic events endured by the young animals. These studies caution the use of skeletochronology for age estimation, especially when applying such methods to the fossil record of vertebrates to deduce age, growth curves, etc.

#### (i) Growth marks in dinosaur bones

Robin Reid was the first to describe the occurrence of zonal bone i.e. periodically interrupted rapid growth, in non-avian dinosaurs [61,62]. He showed that contrary to the reigning ideas at the time, dinosaurs periodically experienced slowed growth and sometimes even stoppages in growth indicated by lines of arrested growth. Since Reid's landmark paper, there have been several other publications that have further demonstrated the widespread occurrence of zonal bone in non-avian dinosaurs [63,64]. Subsequently, Chinsamy *et al.* [65] further demonstrated that the Mesozoic birds, *Patagopteryx* and two enantiornithine birds also experienced such flexible growth patterns. By studying the growth series of two southern African dinosaurs, *Massospondylus* and *Syntarsus*, Chinsamy [64,66] used the growth marks present in the bones of these dinosaurs to estimate the age of the individuals, and then deduced growth curves for them [66,67]. These were the first growth curves ever proposed for dinosaurs. Since these early studies on the osteohistology and ontogenetic growth of non-avian dinosaurs, there has been an explosion of research in this area. Recent studies tend to plot the perimeter of consecutive growth marks against the estimated mass (often derived by using the formulae [68] that are based on the long bone circumference of birds and mammals) [69]. It has also become increasingly evident that given the variation evident within the skeleton of individuals, as well as among similar-sized individuals [70,71], it is imperative that the osteohistology data are carefully constrained for growth curve analysis by considering multiple elements and also standardizing the bones used and regions analysed.

#### (ii) Ontogenetic age assessments in the absence of growth marks

When bone forms in an azonal manner, there is still much information that can be deciphered about the biology of the animal. The nature and texture of the bone tissue can provide information regarding the ontogenetic status of the animal i.e. whether the animal is an embryo, hatchling, juvenile, subadult or an adult. This information is useful in assessing the age structure of an assemblage and can provide clues regarding the association of the material. Embryonic and hatchlings typically have highly porous or spongy bone tissue, indicative of their rapid rate of bone deposition (e.g. [72–74]). In young juveniles, often one can identify a hatchling line (e.g. in egg-laying vertebrates [75,76] or a birth line [37,77] in viviparous mammals). Both these lines mark a change in the rate of bone deposition postnatally. In slightly older juveniles, bone is still rapidly deposited, but the overall tissue becomes more compacted (e.g. [78,79]). With increasingly growth, the depositional rate of bone tissue can change but can still be formed at high rates. As the individuals mature, and possibly when sexual maturity is reached, a change in the rate of bone deposition is often evident, and an overall slowing down in the rate of bone formation occurs, resulting in the deposition of parallel fibred bone or lamellar bone tissues that tend to be more organized and less vascularized (e.g. [9,34]). This often leads to the formation of the outer circumferential layer (OCL); also known as the external fundamental system [80]. In many vertebrates, this tissue marks slow accretional growth, and often rest lines or lines of arrested growth, which indicates periodic cessation of growth can form within this band of tissue (e.g. [50]). Another point worth noting is that during growth and development, the size of the medullary cavity changes, and the endosteal part of the bone often shows secondary remodelling. Once the medullary cavity has completed its expansion, in many vertebrates the deposition of the inner circumferential layer occurs, which lines the medullary cavity (e.g. [81]). Thus, even though growth marks are not present in these vertebrates, one can make reasonable deductions regarding the ontogenetic status of the individuals being studied.

#### (iii) Link between osteohistology and prevailing environmental conditions

The precise driver for the development of growth marks is still uncertain with arguments ranging from unfavourable environmental conditions (e.g. [66,82,83], physiological factors and/or hormonal cues [84]), or internal biorhythm perhaps amplified by seasonal stressors [85,86]. Irrespective of the cause, the mere presence of growth marks in the bones of vertebrates directly indicates a change in the rate of bone deposition, or even cessation of growth (when lines of arrested growth are present). In a carefully constrained study of modern ruminants, Köhler *et al.* [43] showed convincingly that growth is arrested in the unfavourable season together with a decrease in body temperature, metabolic rate, and bone-growth mediating plasma insulin-like growth factor−1 levels, which appear to be linked to a thermometabolic strategy for energy conservation. This study also showed that faster rates of growth are correlated with higher metabolic rates and hormonal changes during the favourable growing season. More recently, Chinsamy & Warburton [38] proposed that the periodic growth marks that occur in the bones of the western grey kangaroo, *Macropus fuliginosus*, were correlated with a thermometabolic strategy (heterothermy), which leads to them decreasing their core body temperatures during the intense heat and dryness of the summer months [87]. Periodic slow down in growth evident in the bones of the desert-dwelling *Addax nasomaculatus* [88] is also considered to have occurred during the unfavourable dry, summer months. Maloney *et al.* [87] postulate that large mammals switch from homeothermy to heterothermy when there is limited water or energy available (i.e. unfavourable season).

Studies of the extinct Miocene and Pleistocene giant thunder birds or mihirungs from Australia show striking growth dynamic patterns that appear to be correlated with the prevailing environments [46,89]: the Miocene *Dromornis stirtoni*, one of the largest known birds to have lived, had the luxury of living under favourable environmental conditions, and took more than a decade to grow up, i.e. a slow life-history strategy. Similar protracted growth rates are known for several island forms, e.g. the elephant bird, *Vorombe titan* [45], several moa taxa [90], *Dinornis* [47], as well as the large Mesozoic bird, *Gargantuavis* [91]. As compared to the Miocene *D. stirtoni*, the Pleistocene *Genyornis* lived at a time of increasing aridity with unpredictability in terms of rainfall, available browse, etc. Under these circumstances, *Genyornis* would have had strong selection pressure to mature faster, and indeed, this is reflected in their bone tissues: only 1–2 growth marks in their bones as opposed to the 14 growth marks evident in their Miocene relatives. Such faster growth rates would have been highly advantageous at the time and would have meant that they would have reached reproductive age sooner. Interestingly, the modern emu was contemporaneous with *Genyornis*, and they had even faster growth rates with no interruptions in their growth. Such rapid growth rates to maturity, combined with the production of large numbers of eggs may have enabled emu to survive the onslaught of humans, which ultimately drove *Genyornis* to extinction [89]. Herein is a caution for the current wave of biodiversity crises: taxa with protracted life histories are at higher risk for extinction under uncertain environmental conditions.

### (b) Palaeoecological impacts of developmental plasticity

Growth plasticity in a population directly impacts the life-history traits of individuals since it affects the attainment of body size, sexual maturity and skeletal maturity with direct consequences for their fitness and fecundity. Thus, assessing whether developmental plasticity occurs in extinct populations can offer significant palaeoecological information.

One of the key ways in which developmental plasticity can be observed in vertebrates is through the observation of growth marks within their bones and changes in the bone tissue pattern [49]. The common occurrence of growth marks in the bones of Mesozoic birds as compared to their neognathous relatives suggests that these early birds exhibited protracted growth strategies as compared to their modern descendants, which typically tend to grow up rapidly within a single year. In a study of Mesozoic mammals, Chinsamy & Hurum [19] proposed that the flexible growth strategies of the Mesozoic eutherians provided an adaptive advantage over the contemporaneous multituberculates during the crisis caused by the end of the Cretaceous extinction event. They postulated that this may have been key to the survival and success of the eutherians following the mass extinction event.

Developmental plasticity is well recognized among many extant vertebrates, such as crocodiles, (e.g. [92]), lizards, (e.g. [93]), birds (e.g. [94]) and mammals (e.g. [86]). Among extinct animals, osteohistology and growth mark analysis have also led to observations of developmental plasticity in sauropodomorph dinosaurs (*Plateosaurus*, Sander & Klein [95]; *Massospondylus* [96], *Mussaurus* [97]), the theropod, *Coelophysis bauri* [98], the ornithischian, *Jehololosaurus shangyuanensis* [99], as well as in the basal beaked bird, *Confuciusornis sanctus* [100].

By studying the number of growth marks in the bones of the Late Triassic, basal sauropodmorph, *Plateosaurus engelhardti*, Sander & Klein [95] found that the histology suggested that different specimens had reached skeletal maturity at different body sizes, i.e. some individuals had attained final body size by 4.8 m in length, whereas others continued to grow until they were about 10 m in length. These findings suggest that individuals in a population can have widely different growth rates and final body size, which led them to suggest that the dinosaurs had variable life histories that most likely were influenced by environmental factors, such as climate and food availability [101]. Although Chinsamy [64,66,67] did not use the term developmental plasticity, she showed that similar-sized femora of the basal sauropodomorph *Massospondylus carinatus* recorded different numbers of growth marks, and more recently, Chapelle *et al.* [96] reported on what they referred to as extreme developmental plasticity in *Massospondylus*. Studies of another sauropodomorph, *Mussaurus* has showed that aside from variation in the number of growth rings present, some individuals showed no growth marks in their bones [97], which highlights the plasticity of the growth dynamics of the taxon, and cautions against the hypothesis of particular growth strategies among basal and derived members of the Sauropodomorpha [102]. Intraspecific variation in histology and growth marks could be the result of many factors, such as sexual dimorphism, localized conditions of growth, and variable response to environmental conditions.

### (c) Insights into gregarious behaviour using growth marks in bone

Besides their use in growth curve analyses, growth marks in vertebrate skeletons have also provided other sorts of ecological information. For example, a monospecific assemblage of *Anikosaurus* showed that all the individuals were similar-aged juveniles, which implied that this taxon exhibited social behaviour [103]. Such juvenile accumulations appear to be relatively common among dinosaurs (e.g. *Psittacosaurus* [104]; *Maiasaurua* [78]). In a recent publication, which also applied osteohistology, Pol *et al.* [105] showed that not only did the basal sauropodomorph *Mussaurus* exhibit gregarious behaviour, but that individuals also segregated according to their age—a behaviour commonly found among large-bodied herbivorous mammals today. They further postulated that such social cohesion and partitioning may have played a role in the first global radiation of sauropodomorph dinosaurs.

More recently, analysis of the humeri of the Triassic archosaur, *Aetosaurus ferratus* [106], suggested that these aetosaur young were gregarious, and that the accumulation reflected a biological/ecological grouping that died in a single catastrophic event. Similarly, Parker *et al.* [107] showed using osteohistology that a group of 12 aetosauriformes *Revueltasaurus callenderi* ranged in age from juvenile to subadult.

### (d) Sex-specific osteohistology

During ovulation, female birds of many species deposit a special bone tissue within the medullary cavities of their bone, which forms a labile store of calcium for eggshell calcification. The actual microscopic and nanoscopic mineral component of medullary bone differs from cortical bone enabling it to cope with the high demands of calcium during oviposition [108]. Thus, the mere presence of this tissue permits the identification of females of a species.

The distribution of medullary bone in the skeleton of reproductive female birds, varies interspecifically, but is affected by pneumaticity and red bone marrow, but curiously appears not to be correlated with clutch size [109]. In general, though it appears to be more widely distributed within the skeletons of small-bodied diving birds but tends to have a more restricted distribution within the skeletons of large-bodied taxa or efficient flyers [109].

The identification of females is particularly important for palaeoecological deductions among extinct animals for which there are generally few cues regarding the sex of an individual. Schweitzer *et al.* [110] identified some unusual bone tissues within the medullary cavity of *Tyrannosaurus rex* and postulated that this was homologous to the avian medullary bone, and that the *T. rex* was a female individual. This was quite a novel finding and inspired many other palaeontologists to search for such ‘medullary bone’ in other dinosaurs. Some identifications of ‘medullary bone’ were made (e.g. *Allosaurus* and *Tenontosaurus* [111]; *Dysalatosaurus* [112]; *Mussaurus* [113]; and an ornithomimid from Uzbekistan [114]). However, it also became apparent that not all bone within the medullary cavity is homologous to the avian medullary bone. It turns out that pathological bone can also develop from the endosteal margin of the bone wall, and can look very similar to so-called medullary bone tissues [39]. Indeed, the *Allosaurus* specimen in which medullary bone was identified (Lee & Werning [111]) also shows a periosteal pathological bone tissue, and the *Mussaurus* specimen that Cerda & Pol [113] described was later considered to be pathological [115]. Tremaine *et al.* [116] described a medullary bone-like tissue within the marrow cavity of a juvenile *Tyrannosaurus*, which suggests that this tissue is pathological. Various biochemical methods have been tested to distinguish between endosteally formed pathological bone and medullary bone, but to date, there is not a reliable way to distinguish between these tissues [117].

**Figure 2.**
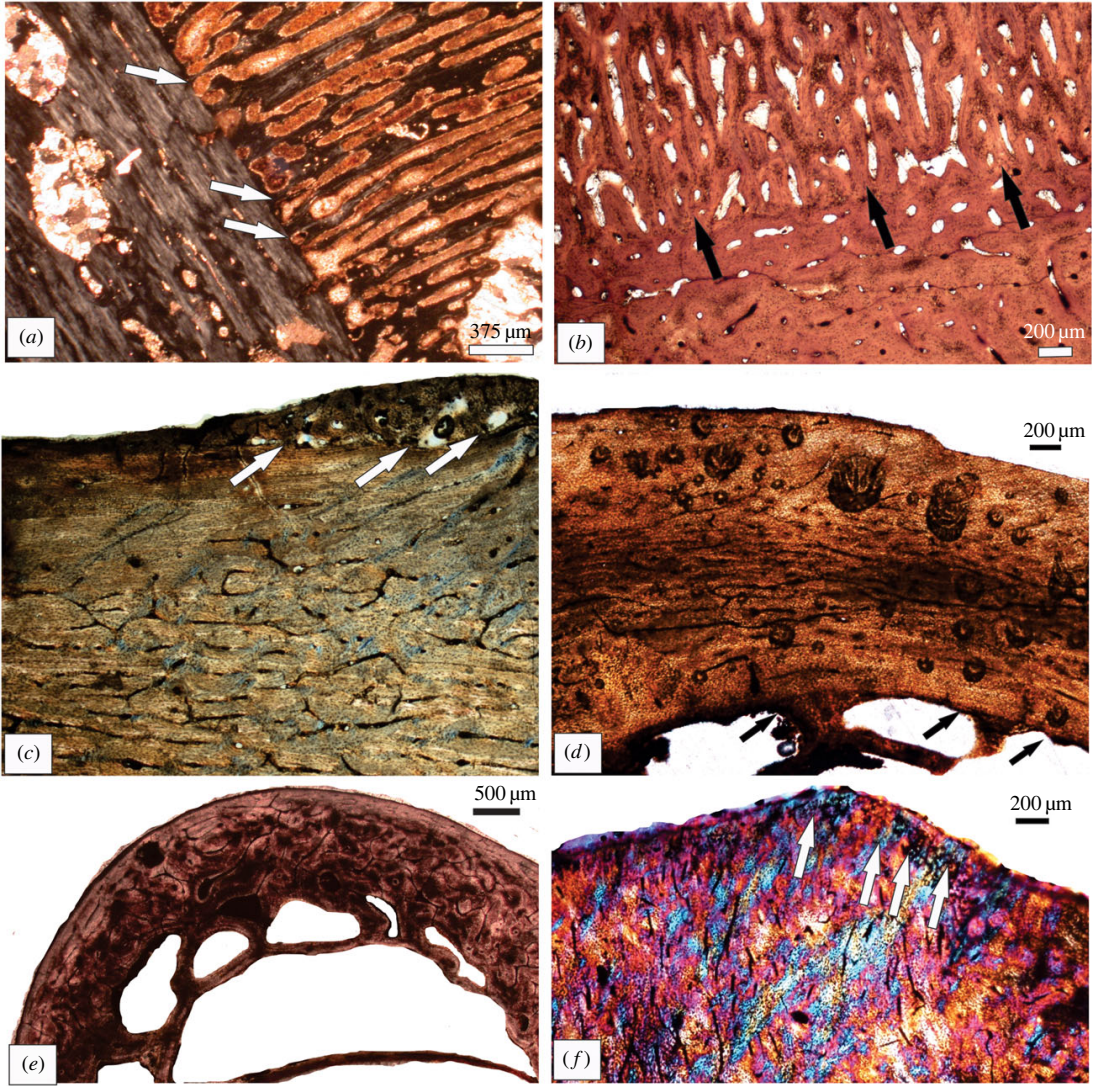
Periosteal reactive growth (arrows) in (*a*) Transylvanian dinosaur long bone, R5505, (*b*) tibia of the *Stegosaurus* sp., YPM 57509, (*c*) *Gastornis* tibiotarsus (tb-1b), (*d*) medullary bone (arrows) in a tibiotarsus of a dodo, (*e*) osteoporotic cavities caused by moulting in *Spheniscus demersus*, and (*f*) fibrocartilage enthesis (arrows) in the femur of *Macropus fuliginosus* (KNW7).

The identification of medullary bone in Mesozoic birds began with the tissue first being recognized in *Confuciusornis* [118], and has since been identified in a few other enantiornithine taxa (a pengornithid [119], *Miruavis* [120]) and *Avimaia* [121])*.* Medullary bone has also been described in the recently extinct dodo (figure 2*e*) [122].

#### (i) Sex related differences in skeletal homeostasis

Although it is well recognized that age-related bone loss (osteopenia, which is a pre-cursor to osteoporosis) is quite common among mammals, there are very few studies that have demonstrated differences in mineral mobilization between males and females of the same species.

Hutton [92] reported that the cortical structure of osteoderms in breeding female crocodiles showed an abundance of erosion cavities as compared to males, which he attributed to the mobilization of calcium for egg shelling. Similar findings have been documented for alligators [123–126].

Among mammals, a recent study of *B. suillus*, the modern Cape dune mole rat, Montoya-Sanhueza & Chinsamy [20] found quantifiable differences between males and females in terms of secondary reconstruction. Although secondary reconstruction occurred in both sexes, only females showed subendosteal secondary reconstruction. Also, in contrast to males that show smaller more randomly distributed erosion cavities, females exhibited several secondary enlarged erosion cavities in the perimedullary region which indicated a sixfold increase in bone loss as compared to males [20]. Based on these findings, it is proposed that *Bathyergus* shows sexual dimorphism in terms of mineral homeostasis, like many other mammalian species. Interestingly a study on lactating female naked mole-rats, *Hetreocephalus glaber* suggested an increase in the number of resorption cavities in their bones [127].

### (e) Response to trauma and disease

Often when bones are studied anatomically, unusual pathological features, such as fracture calluses, or arthritis are easily notable (e.g. [128]). However, there are many studies that have documented other types of skeletal pathologies in extant and extinct animals based on morphological observations (figure 2*a–c*), e.g. [129–133]. Indeed, some diseases simply do not manifest on the bone surface or the animal had not lived long enough for the condition to alter the exterior bone morphology, but histological examination sometimes can suggest a pathology. Then there are instances where the gross anatomy can suggest a particular kind of trauma, but at a histological level, the nature of the pathology can be better diagnosed (e.g. [77,134,135]). Infections or osteomyelitis has a fairly distinctive character and has been identified microscopically in many different taxa, e.g. in *Stegosaurus* [136] (figure 2*b*), as well as in a Permian dinocephalian [137]. There have been several examples of osteosarcoma reported in fossil bones based on osteohistological diagnoses, e.g. in a Triassic-aged turtle [138], Pliocene seal (Woolley *et al.* [77]) and periostitis has been diagnosed osteohistologically in a Permian gorgonopsian [139]. Chinsamy & Turmarkin-Deratzian [39] reported on presumed avian osteopetrosis in a Transylvanian dinosaur (figure 2*a*) which compared well with the histological features evident in a modern turkey vulture afflicted with the same disease.

### (f) Skeletal response to moulting

All birds undergo an annual moult, which can be sequential, simultaneous, or catastrophic [140]. The regrowth of feathers poses a high demand for calcium, which is removed from their bones and results in osteoporotic bones (figure 2*e*) [141]. This is particularly true of birds like penguins that undergo a catastrophic moult. As early as 1951, Meister [141] showed that the bones of 15 bird species (including penguins) changes during the moult cycle: their bones become extremely porous during the moult because of the increased calcium demand on the skeleton, and after the moult, the large erosion spaces become secondarily infilled. Murphy *et al.* [142] found that the occurrence of erosion cavities in the tarsometatarsi and tibiotarsi of white-crowned sparrows was linked to moulting and a decreased body weight. The presence of large erosion cavities in the bones of a moulting African penguin (figure 2*e*), *Spheniscus demersus* was reported by Dabee [143]. In addition, Whyte [144] reported similar osteoporotic cavities in moulting Egyptian geese, and she showed that the peak resorption occurred when the new feathers were two-thirds fully grown.

In terms of the fossil record, Cerda *et al.* [33] ascribed the highly porous bone of an Antarctic Eocene penguin to having died during its annual moult. In addition, the presence of large erosion cavities in the bones of the dodo, *Raphus cucullatus*, were reasoned to have been formed during moulting [122], and these data were used to reconstruct various aspects of the ecology and life history of the dodo.

### (g) Investigating ecomorphological adaptations through osteohistology

#### (i) Filter feeding adaptations in *Pterodaustro*

Histological studies have highlighted the extraordinary ecomorphological adaptations evident in the teeth of the Early Cretaceous pterosaur, *Pterodaustro guinui.* It has the distinction of having rather unusual dentition as compared to other pterosaurs: its lower jaw is lined with more than a thousand long, thin (less than 1 mm thick), filamentous structures, that Chiappe & Chinsamy [145] showed were modified true teeth, complete with dentine and enamel, that are used for its filter-feeding lifestyle.

More recently, Cerda & Codorniú [146] investigated how *Pterodaustro*'s filter-feeding teeth were implanted into the lower jaw. They found that the teeth were positioned in a groove without any interdental separation between them, i.e. neighbouring teeth had no spaces between them, which is thought to have aided its filtering strategy by preventing the loss of any filtered material. Since these researchers were also unable to identify any replacement teeth, they have proposed that rather unusually, this filter-feeding pterosaur had monophyodonty or diphyodonty. This is unexpected for an archosaur, but so is the fact that no gomophosis is evident (i.e. no cementum nor periodontal ligaments or alveolar bone was present). Interestingly, ankylosis could also not be confirmed in this unusual pterosaur. Thus, it appears that the modified filter-feeding dentition in *Pterodaustro* [145] was also unique in the way in which the teeth were implanted in the jaw [146]. It is likely that these features in *Pterodaustro*, which deviate from typical archosaur morphology may reflect special adaptations for its peculiar filter-feeding lifestyle.

#### (ii) Mechanosensory structures within the jaws of probe feeders

Some probe-feeding birds locate their prey by detecting vibrations in the sediment or water by using unique mechanosensory structures (Herbst corpuscles) located within bony pits in the tips of their jaws [147]. By undertaking a thorough examination of the histology of the tips of the beaks of extant probing birds, these researchers showed that the actual morphology of the bony beak i.e. the ratio of the beak–skull length, and the number and concentration of the bony pits at the tip of the beak, can be used as an osteological correlate to infer the presence of Herbsts corpuscles in extinct bird taxa to provide information about their palaeoecology. It is notable that they were able to successfully deduce the presence of a bill tip organ (without soft tissue data) in 353 extant species representing 200 families [147]. Applying these specially developed methods to the common ancestor of all palaeognathous birds, the lithornithids, Du Toit *et al.* [147] showed that these Cretaceous birds were quite likely probe foragers. These findings explain the enigma of why some non-kiwi ratites such as ostriches, emu, cassowary, etc. have a similar bill tip organ (in the absence of a probe feeding lifestyle): it is a retention of a plesiomorphic trait. Thus, here we see how histological investigations combined with morphological studies have permitted deductions that this sophisticated remote touch sensory system had evolved in the Cretaceous already, and similarities with the anseriform bill tip organ suggests that this may have been well before the palaeognathous–neognathus split [147].

#### (iii) Fibrocartilage enthesis linked to saltatorial locomotion

In a study of ontogenetic growth in the western grey kangaroo, *Macropus fuliginosus*, Chinsamy & Warburton [38] reported on the specialized enthesis on the caudal tuberosity of the femur for the attachment of m. quadratus femoris. Since the function of an enthesis prevents avulsion by dissipating stresses at the tendon–bone interface, it is likely that this feature develops in kangaroos as a biomechanical adaptation in response to their saltatorial lifestyle. It is suggested that the presence or absence of a fibrocartilage enthesis in the femora of extinct macropods could indicate whether they employed a saltatorial locomotory habit.

## 4. Concluding remarks

Osteohistology is a powerful tool to unravel the biology of extinct animals and helps to understand them within the context of their ecology. However, it is limited in that because of the destructive nature of the methodology, it generally involves small sample sizes, and often just a core or one or a few serial sections of a skeletal element. Non-invasive methods such as CT scanning and synchrotron scanning circumvent the issue of destructive analyses, and permit the visualization of virtual histology, but currently, these methodologies are expensive and not readily accessible. Furthermore, although they are useful in providing a three-dimensional visualization of the spatial architecture, the actual tissue-level organization of the bone is generally not visible (e.g. [148]). Submicron, higher resolution can sometimes be obtained from small enough specimens, but whether these are reliable for extrapolation to the whole bone is uncertain (e.g. [148]). However, perhaps even newer technological advances will further enhance our assessment of the three-dimensional microstructural organization of bones, and perhaps even overcome the current limitations.

Consummate with the growth of palaeohistology, gaps in our knowledge of modern bone biology have become evident, and as a result there has been a correlated upsurge of osteohistology work on modern vertebrates. This is especially important since it is starkly evident that sound palaeoecological interpretations must be based on rigorous data derived from modern ecological systems.

## Data Availability

This article has no additional data.
